# Quantifying rural disparity in healthcare utilization in the United States: Analysis of a large midwestern healthcare system

**DOI:** 10.1371/journal.pone.0263718

**Published:** 2022-02-10

**Authors:** Akua Nuako, Jingxia Liu, Giang Pham, Nina Smock, Aimee James, Timothy Baker, Laura Bierut, Graham Colditz, Li-Shiun Chen

**Affiliations:** 1 Department of Psychiatry, Washington University School of Medicine, St. Louis, MO, United States America; 2 Alvin J. Siteman Cancer Center at Barnes-Jewish Hospital, Washington University School of Medicine, St. Louis, MO, United States America; 3 Department of Medicine, University of Wisconsin School of Medicine and Public Health, Madison, WI, United States America; 4 Division of Public Health Sciences, Department of Surgery, Washington University School of Medicine, St. Louis, MO, United States America; University of Arkansas for Medical Sciences, UNITED STATES

## Abstract

**Purpose:**

The objective of this study is to identify how predisposing characteristics, enabling factors, and health needs are jointly and individually associated with epidemiological patterns of outpatient healthcare utilization for patients who already interact and engage with a large healthcare system.

**Methods:**

We retrospectively analyzed electronic medical record data from 1,423,166 outpatient clinic visits from 474,674 patients in a large healthcare system from June 2018-March 2019. We evaluated patients who exclusively visited rural clinics versus patients who exclusively visited urban clinics using Chi-square tests and the generalized estimating equation Poisson regression methodology. The outcome was healthcare use defined by the number of outpatient visits to clinics within the healthcare system and independent variables included age, gender, race, ethnicity, smoking status, health status, and rural or urban clinic location. Supplementary analyses were conducted observing healthcare use patterns within rural and urban clinics separately and within primary care and specialty clinics separately.

**Findings:**

Patients in rural clinics vs. urban clinics had worse health status [χ^2^ = 935.1, df = 3, p<0.0001]. Additionally, patients in rural clinics had lower healthcare utilization than patients in urban clinics, adjusting for age, race, ethnicity, gender, smoking, and health status [2.49 vs. 3.18 visits, RR = 0.61, 95%CI = (0.55,0.68), p<0.0001]. Further, patients in rural clinics had lower utilization for both primary care and specialty care visits.

**Conclusions:**

Within the large healthcare system, patients in rural clinics had lower outpatient healthcare utilization compared to their urban counterparts despite having potentially elevated health needs reflected by a higher number of unique health diagnoses documented in their electronic health records after adjusting for multiple factors. This work can inform future studies exploring the roots and ramifications of rural-urban healthcare utilization differences and rural healthcare disparities.

## Introduction

Rural populations, who make up around ~19% of the United States’ population [1, 2], have heightened mortality rates from several chronic diseases relative to urban populations [2]. The rural-urban mortality disparity has grown over recent decades despite overall declines in mortality from such conditions [3]. The causes of rural health outcome disparities are multiple and complex, but importantly include higher rates of poverty and behavioral risk factors, larger proportions of elderly individuals and individuals with disabilities, lower rates of insurance and education, and limited access to healthcare providers and healthcare encounters in rural regions [4–8]. Given rural communities’ amplified barriers to acquiring healthcare, further quantification of differences in healthcare utilization across rural and urban populations using healthcare systems data can potentially provide important insight into rural health inequities among patients engaged in healthcare systems.

Healthcare utilization is a broad term that describes individual or population-level use of healthcare services delivered in outpatient facilities, hospitals, or homes [9, 10]. While individual patterns of healthcare utilization may vary based on numerous factors [11], there are existing comprehensive theoretical models such as Andersen’s Behavioral Model of Health Services Use that predict healthcare utilization based on predisposing characteristics, enabling factors, and health needs [12]. Therefore, when studying utilization trends across population groups, it important to also account for predisposing characteristics such as sociodemographic and behavioral factors that have been found to be associated with use of healthcare utilization. For example, a higher proportion of rural residents have no healthcare visits in a year compared to urban residents [13]. Additionally, younger people, Black people, and males have fewer physician’s office visits in a year than older people, White people, and females, respectively [14–17]. Furthermore, people who smoke have been shown to have higher overall outpatient healthcare utilization despite using preventive healthcare services less frequently than people who do not smoke [18–22].

There are a few reasons that highlight the need for quantifying rural disparity in healthcare utilization using real world healthcare system data. First, healthcare system data represents a complementary population to general population survey data. Much of the existing evidence on healthcare utilization has been based on general population surveys or Medicare fee-for-service claims data [23–25]. It is possible that general population surveys on healthcare utilization may be largely impacted by responses from people with few healthcare needs relative to people who interact with the healthcare system. In fact, nearly 16% of respondents to the National Health Interview Survey (NHIS), a prototypical general population survey examining health behaviors and outcomes, have no healthcare encounters in a year [26]. While this could partially be due to a relative lack of healthcare access amongst this group, NHIS surveys have been found to have a bias for healthier patients relative to the general public [27]. The Medicare-based studies may also be non-representative of the overall adult population given their focus on a population in which adults age 65+ are overrepresented. Second, healthcare system data will allow us to not only quantify the differences between healthcare utilization patterns in patients in rural and urban clinics, but also to adjust for additional predictors like individual health status and smoking status that are potentially associated with utilization. This methodology can attenuate the effects of confounding factors that could impact national data given that the study populations could come from different healthcare systems or structures. It is important to examine whether factors observed in existing studies based on surveys and Medicare claims data hold true among a general adult population who accesses care within the same large healthcare system. To further current evidence on rural health disparity based on national survey or claims data, our study focuses on a more homogeneous population from a single large healthcare system among patients with existing access to healthcare, and uses real-world EHR data to derive utilization and health status, filling a gap on disparity among patients accessing the same healthcare system.

The objective of this study is to identify how predisposing characteristics, enabling factors, and health needs are jointly and individually associated with epidemiological patterns of outpatient healthcare utilization for patients who already interact and engage with a healthcare system. We hypothesize that, among this patient population, healthcare utilization among those accessing care will be lower in patients in rural versus urban clinics, even after adjusting for other significant predictors.

## Materials and methods

### Design and setting

This study used a cross-sectional, retrospective design to examine the level of outpatient healthcare utilization among patients serving the greater St. Louis, southern Illinois, and mid-Missouri regions stratified by age, gender, race, ethnicity, smoking status, health status and rural versus urban clinic location. Data used for these analyses included electronic health records (EHR) for outpatient visits from 2 June 2018 to 31 March 2019, a timeframe that encompassed the first ten months of the healthcare system’s use of the EHR system observed in this study. Of the 766 included clinics, 693 were classified as urban and 73 were classified as rural.

### Population

Our healthcare system provided care for 474,674 adult patients aged 18 years and older with 1,423,166 documented in-person outpatient clinical evaluation and management encounters. For this study to evaluate patients from rural vs. urban clinics, we evaluated 124,688 rural outpatient visits (from 50,250 patients who visited only rural clinics) and 1,269,975 urban outpatient visits (from 424,424 patients who visited only urban clinics). For the purpose of comparing patients in rural vs. urban clinics, we excluded a small portion of 28,503 outpatient visits (from 4,456 patients who visited both urban and rural clinics).

### Variables

**Fig 1:**
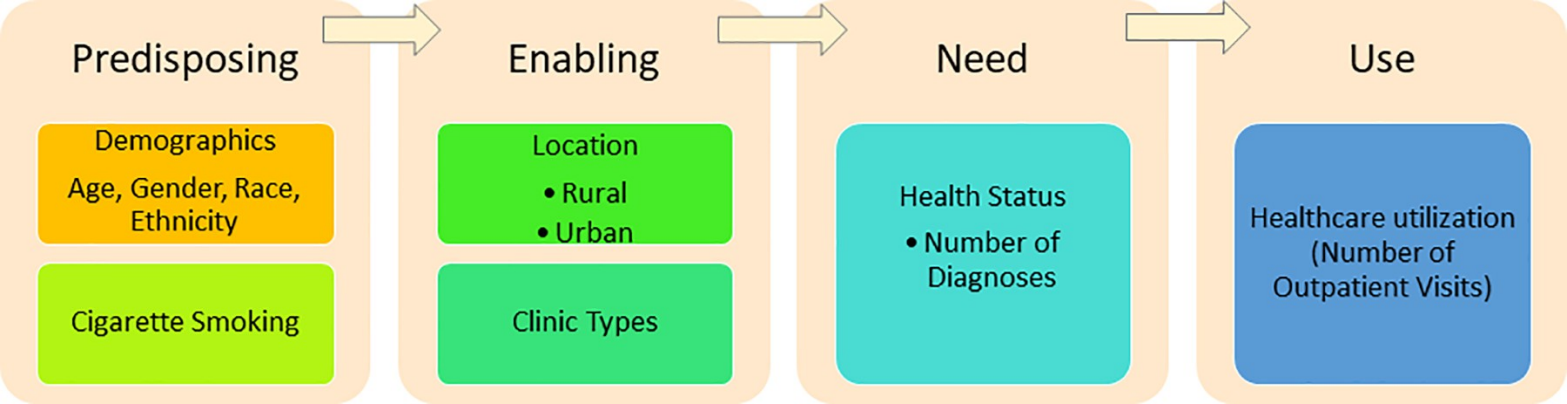
Adapted Andersen Behavioral Model of Health Services Use.

The number of outpatient visits during the study period was the outcome variable chosen as a proxy for level of outpatient healthcare utilization. The independent variables were selected based on several “predisposing characteristics” from an adopted Andersen model of healthcare utilization that were consistently documented and accessible within the EHR for the majority of patients [12]. These included several predisposing characteristics (age, gender, race, ethnicity, cigarette smoking), enabling factors (rural or urban clinic location, clinic types), and health need (health status) (See Fig 1).

#### Age, gender, race, and ethnicity

Information on age (coded as 18–49, 50–59, 60–69, ≥70), gender (male or female), race (White, Black, or Other), and ethnicity (Hispanic or Non-Hispanic) were obtained in the EHR.

#### Cigarette smoking

If an individual was ever documented as a person who smoked in a clinic encounter examined within the data timeframe, they were classified as a person who smoked. This identity was self-reported at the time of clinic encounter.

#### Location

We categorized rural versus urban location using clinic instead of patient home addresses in order to more directly examine the setting in which outpatient clinical encounters occurred. To categorize clinics by location, we input clinic address into Google Earth Pro and classified the clinic’s county into one of six levels based on the CDC’s 2013 National Center for Health Statistics Urban–Rural Classification Scheme for Counties. Following this guideline, metropolitan (“urban”) clinics included the first four levels of the Metropolitan Statistical Areas (large central metro, large fringe metro, medium metro, and small metro), while non-metropolitan (“rural”) clinics included those in micropolitan and noncore statistical areas [28].

#### Health status

Health status was determined by the number of unique diagnoses in the EHR data, and was converted into quartiles in analyses (≤2, 3–5, 6–8, and ≥9 diagnoses). The number of diagnoses is used as the measure for patient health status adapted from other studies [29–31]. We also used an alternative measure of comorbidity, the Elixhauser comorbidity score in additional analyses [32].

#### Clinic type

In secondary analyses, we evaluated rural disparity in primary vs. specialty clinics. Primary care clinics included Family Medicine, Internal Medicine, and Pediatrics. All other clinics such as Ancillary Services, Dermatology, Surgery Departments, Obstetrics & Gynecology, Ophthalmology, Pain Management, Physical Medicine and Rehabilitation, Psychiatry, Radiation Oncology and Radiology were considered specialty clinics, see **S1 Table** for the full list of clinics.

#### Health professional shortage

In secondary analyses, we defined level of health professional shortage in each clinic location by obtaining data from the Area Health Resource File (AHRF) from data.HRSA.gov [7] and dividing counties into tertiles (low, medium, high).

### Statistical analysis

The descriptive statistics were provided for the primary outcome and covariates. The univariate and multivariate generalized estimating equation (GEE) models with Poisson link function were employed to investigate the effect of the covariates on healthcare utilization, in which the correlation among patients within clinics was considered. Relative risks (RRs) and 95% CIs were estimated and the standard errors were calculated using the GEE sandwich method when accounting for within-clinic correlation. The comparisons of the distributions of predisposing characteristics, enabling factors and health needs were conducted through χ^2^ tests. Supplementary analyses were conducted observing healthcare utilization patterns within rural and urban clinics separately and primary care and specialty clinics separately. To address the concern of multiple comparisons, we adjusted our significance threshold from 0.05 to 0.001 given the number of tests conducted. All analyses were conducted using R version 3.5.3 for Microsoft Windows and SAS software, Version 9.4m6 of the SAS system for Microsoft Windows.

### Ethics statement

The Institutional Review Board determined this study involved non-human subjects and waived the requirement for informed consent because this is part of conducting a quality improvement project. This study did not meet federal definitions under the jurisdiction of the Institutional Review Board and falls outside the purview of the Human Protection Office.

## Results

### Study sample

Our sample included a total of 474,674 patients. The combined patient sample included 59.5% females, 80.1% White patients, 15.7% Black patients, 98.5% Non-Hispanic patients, and 14.7% patients who smoke, and 10.6% patients in exclusively rural clinics (**Table 1**).

**Table 1:** Sample characteristics^a^.

	(N = 474,674)
	n (%)
Age (years)	
18–49	171,566 (36.1%)
50–59	91,079 (19.2%)
60–69	105,622 (22.3%)
≥70	106,407 (22.4%)
Gender^b^	
Male	192,197 (40.5%)
Female	282,283 (59.5%)
Race^c^	
White	371,208 (80.1%)
Black	72,575 (15.7%)
Other	19,273 (4.2%)
Ethnicity^d^	
Hispanic	6,493 (1.5%)
Non-Hispanic	440,672 (98.5%)
Smoking Status^e^	
Non-smoker	360,661 (85.3%)
Smoker	62,120 (14.7%)
Clinic location^f^	
Urban	424,424 (89.4%)
Rural	50,250 (10.6%)
Health Status^g^	
Q1 (≤2 diagnoses)	143, 702 (30.3%)
Q2 (3–5 diagnoses)	102,795 (21.7%)
Q3 (6–8 diagnoses)	126,909 (26.7%)
Q4 (≥9 diagnoses)	101,268 (21.3%)

^a^ The sample was from outpatients, serving the greater St. Louis, southern Illinois, and mid-Missouri regions from June 2018- March 2019.

^b^ Documented gender was missing for 194 patients.

^c^ Documented race was missing for 11,618 patients.

^d^ Documented ethnicity was missing for 27,509 patients.

^e^ Smoking status was missing for a total of 51,893 patients.

^f^ These patients are unique and exclusively visited urban or rural clinics.

^g^ Health Status is defined as the number of ICD 10 diagnosis codes by quartile.

### Patient characteristics in rural vs. urban clinics

The characteristics of the rural and urban samples are shown in **S2 Table**. Compared to the urban group, notable differences in the rural group include a higher proportion of White patients (94.4% vs 78.5%, χ^2^ = 7608, df = 2, p<0.0001), a higher proportion of patients who smoke (20.7% vs 13.9%, χ^2^ = 1504, df = 1, p<0.0001), and a higher proportion of patients with worse health status defined by more diagnoses (overall χ^2^ = 935.1, df = 3, p<0.0001).

#### Healthcare utilization across patients characteristics

The number of outpatient clinic visits across age, gender, race, ethnicity, smoking status, and health status groups are shown in **Table 2**, showing variable utilization across age, gender, race, smoking, and health status groups. Healthcare utilization across these patient characteristics in is also shown in **Table 3**, showing a general pattern of lower utilization in patients seen in rural vs. urban clinics.

**Table 2:** Healthcare utilization across patient characteristics^a^.

	*Average number of visits*
	*mean (sd)* (*N* = 474,674)
Age	
18–49	2.57 (2.59)
50–59	3.04 (3.15)
60–69	3.41 (3.71)
≥70	3.71 (3.94)
Gender	
Male	3.08 (3.46)
Female	3.12 (3.24)
Race	
White	3.08 (3.33)
Black	3.46 (3.51)
Other	2.70 (2.82)
Ethnicity	
Hispanic	2.96 (3.02)
Non-Hispanic	3.17 (3.39)
Smoking Status^b^	
Non-smoker	3.09 (3.34)
Smoker	3.21 (3.28)
Location^c^	
Urban	3.18 (3.38)
Rural	2.49 (2.84)
Health Status^d^	
Q1(≤2 diagnoses)	1.51 (1.48)
Q2 (3–5 diagnoses)	2.08 (1.85)
Q3(6–8 diagnoses)	2.44 (2.85)
Q4 (≥9 diagnoses)	6.15 (4.58)

^a^ Healthcare Utilization is defined as number of visits to any outpatient clinics in 766 clinics serving the greater St. Louis, southern Illinois, and mid-Missouri regions from June 2018- March 2019.

^b^ Individuals were classified as smokers in this study if they were ever documented as a smoker in a clinic encounter recorded within the data timeframe. This identity was self-reported at the time of clinic encounter.

^c^ These patients are unique and exclusively visited urban or rural clinics.

^d^ Health Status is defined as the number of ICD 10 diagnosis codes by quartile.

**Table 3:** Healthcare utilization across patient characteristics in rural vs. urban clinics^a^.

	Overall	Rural	Urban
	*Average number of visits*	*Average number of visits*	*Average number of visits*
	*mean (sd)*	*mean (sd)*	*mean (sd)*
Age			
18–49	2.57 (2.59)	2.12 (1.74)	2.64 (2.68)
50–59	3.04 (3.15)	2.37 (2.38)	3.11 (3.22)
60–69	3.41 (3.71)	2.64 (3.19)	3.50 (3.75)
≥70	3.71 (3.94)	3.10 (4.00)	3.79 (3.92)
Gender			
Male	3.08 (3.46)	2.46 (3.14)	3.16 (3.49)
Female	3.12 (3.24)	2.52 (2.62)	3.20 (3.30)
Race			
White	3.08 (3.33)	2.52 (2.87)	3.17 (3.39)
Black	3.46 (3.51)	2.35 (2.60)	3.48 (3.52)
Other	2.70 (2.82)	2.04 (2.14)	2.75 (2.86)
Ethnicity			
Hispanic	2.96 (3.02)	2.46 (2.45)	3.00 (3.06)
Non-Hispanic	3.17 (3.39)	2.54 (2.90)	3.25 (3.43)
Smoking Status^b^			
Non-smoker	3.09 (3.34)	2.48 (2.93)	3.16 (3.37)
Smoker	3.21 (3.28)	2.57 (2.48)	3.33 (3.39)
Health status^c^			
Q1(≤2 diagnoses)	1.51 (1.48)	1.46 (2.39)	1.52 (1.34)
Q2 (3–5 diagnoses)	2.08 (1.85)	1.71 (1.47)	2.12 (1.88)
Q3(6–8 diagnoses)	2.44 (2.85)	2.15 (1.80)	2.95 (2.51)
Q4 (≥9 diagnoses)	6.15 (4.58)	4.69 (3.89)	6.34 (4.62)

^a^ Healthcare Utilization is defined as number of visits to any outpatient clinics in 766 clinics serving the greater St. Louis, southern Illinois, and mid-Missouri regions from June 2018- March 2019.

^b^ Individuals were classified as smokers in this study if they were ever documented as a smoker in a clinic encounter recorded within the data timeframe. This identity was self-reported at the time of clinic encounter.

^c^ Health Status is defined as the number of ICD 10 diagnosis codes by quartile.

First, most demographic, smoking, and health status factors were associated with utilization in univariate analyses. Specific results of univariate analyses from a GEE model that tests the association of health need and several predisposing characteristics and enabling factors with healthcare utilization are shown in **Table 4** left panel. Older age was associated with increasing visits [age ≥70 vs. age 18–49, RR = 1.37, 95%CI = (1.33, 1.41); p<0.0001]. Female vs. male gender was also associated with higher utilization [RR = 1.04, 95%CI = (1.03, 1.06), p<0.0001]. Black vs. White patients had higher utilization [RR = 1.06, 95%CI = (1.05, 1.08), p<0.0001] and patients from other racial groups had lower utilization [RR = 0.89, 95%CI = (0.87, 0.92), p<0.0001]. Smoking was associated with higher utilization [RR = 1.04, 95%CI = (1.02, 1.05), p<0.0001]. Worse health status was associated with higher utilization [Q4 vs. Q1, RR = 4.51, 95%CI = (4.25, 4.78); p<0.0001]. Additionally, we noted a trend of lower utilization in patients in rural vs. urban clinics that has not reached statistical significance in univariate analyses. Second, we found that rural location and health status were significant predictors for utilization in multivariate analyses when adjusting for demographic factors and all predictors (Table 4 right panel). The multivariate analyses demonstrated a lower utilization among patients in rural vs. urban clinics [2.49 vs. 3.18 visits, RR = 0.61, 95%CI = (0.55, 0.68), p<0.0001] (Tables 3 and 4). Health status remained a significant predictor for utilization [Q4 vs. Q1, RR = 4.52, 95%CI = (4.26, 4.79); p<0.0001]. Age remained a significant predictor for utilization. However, the associations of gender, race, and smoking with level of healthcare utilization observed in univariate analyses were no longer significant after controlling for the other variables in the multivariate model. In additional analyses, we reached similar results when adjusting for additional covariates such as the Elixhauser comorbidity score [32] and health professional shortage as shown in **S3 and S4 Tables**.

**Table 4:** Association of patients characteristics and healthcare utilization: Univariate and multivariate analyses.

	Univariate Analysis			Multivariate Analysis		
	RR^b^	95% CI	p*	RR	95% CI	p
Age						
18–49	Reference			Reference		
50–59	1.13	1.12, 1.15	**<0.0001**	0.97	0.96, 0.98	**<0.0001**
60–69	1.26	1.24, 1.28		0.99	0.98, 1.00	
≥70	1.37	1.33, 1.41		1.02	1.00, 1.03	
Gender						
Male	Reference			Reference		
Female	1.04	1.03, 1.06	**<0.0001**	0.99	0.98, 1.00	0.307
Race						
White	Reference			Reference		
Black	1.06	1.05, 1.08	**<0.0001**	1.00	0.99, 1.01	0.207
Other	0.89	0.87, 0.92		0.99	0.97, 1.00	
Ethnicity						
Hispanic	0.96	0.94, 0.99	0.031	1.01	1.00, 1.03	0.18
Non-Hispanic	Reference			Reference		
Smoking Status^c^						
Non-smoker	Reference			Reference		
Smoker	1.04	1.02, 1.05	**<0.0001**	1.00	0.99, 1.01	0.76
Health status^d^						
Q1(≤2 diagnoses)	Reference			Reference		
Q2 (3–5 diagnoses)	1.68	1.61, 1.76	**<0.0001**	1.69	1.62, 1.76	**<0.0001**
Q3(6–8 diagnoses)	2.40	2.27, 2.53		2.40	2.27, 2.54	
Q4 (≥9 diagnoses)	4.51	4.25, 4.78		4.52	4.26, 4.79	
Location^e^						
Urban	Reference			Reference		
Rural	0.93	0.70, 1.23	0.594	0.61	0.55, 0.68	**<0.0001**

^a^ Healthcare Utilization is defined as number of visits to any outpatient clinics in 766 clinics serving the greater St. Louis, southern Illinois, and mid-Missouri regions from June 2018- March 2019.

^b^ RR: Relative risk.

^c^ Individuals were classified as smokers in this study if they were ever documented as a smoker in a clinic encounter recorded within the data timeframe. This identity was self-reported at the time of clinic encounter.

^d^ Health Status is defined as the number of ICD 10 diagnosis codes by quartile.

^e^ These patients are unique and exclusively visited urban or rural clinics.

*Boldface indicates statistical significance (p<0.001).

**Fig 1** visually demonstrates our adapted Andersen Behavioral Model of Health Services Use with the predisposing characteristics, enabling factors, and health needs that were considered in our study (Fig 1).

### Medical specialties of clinics by location

The distribution of primary vs. specialty clinics also differ in rural vs. urban settings as shown in **S1 Table.** “Primary care” clinic types included Family Medicine, Internal Medicine, and Pediatrics clinics while “specialty” clinics included those from all other specialties. There were notably fewer clinics in rural areas [73 rural clinics (9.5%); 693 urban clinics (90.5%)]. Primary care clinics made up a significantly higher proportion of clinics in rural areas compared to urban areas (71% of rural clinics; 44% of urban clinics) and specialty clinics were more prevalent in urban areas.

### Healthcare utilization across patients characteristics: Primary vs. specialty care

Given the predominance of primary care clinics and the relative lack of specialty clinics in rural areas, we evaluated if rural disparity in utilization was observed in in primary care vs. specialty clinic types (**S5 and S6 Tables**). We observed a similar trend of rural disparity in utilization across primary and specialty care clinics.

## Discussion

Using data from a large healthcare system, we have evaluated patient characteristics and healthcare utilization in rural vs. urban clinics to better understand the extent and pattern of health disparity. We present evidence to quantify a significantly lower utilization of outpatient care among patients in rural clinics, even after adjusting for health status and several predisposing characteristics and enabling factors.

First, we confirmed prior findings that predisposing demographic factors are associated with utilization. Older patients had higher utilization, even considering other predictors such as health status. This could be due in part to the high prevalence of Medicare coverage above age 65 minimizing barriers to healthcare access caused by lack of insurance. In addition, we also identified the impact of race, gender, and smoking on utilization; with Black race, female gender, and cigarette smoking associated with higher utilization. However, these associations were likely mediated through individual’s health status as they became insignificant when adjusting for other factors. We have conjectures as to why these populations might have higher health needs based on health status, including that Black populations face higher burdens of several diseases than do White populations [33], obstetric and/or gynecological care recommendations for female patients may increase their prescribed health needs relative to men, and smoking is associated with a number of health consequences that lead to increased health needs.

Second, we found lower healthcare utilization among rural clinic patients compared to their urban counterparts, with 22% fewer interactions with the healthcare system on average than urban clinic patients over the study period. Adjusting for other variables (age, gender, race, ethnicity, smoking status, and health status), patients in rural vs. urban clinics were associated with significantly less outpatient visits. This rural disparity becomes more apparent when multiple predictors for utilization are also considered. For example, our findings shows that rural vs. urban clinic patients were more likely to be smokers (20.7% vs 13.9%) with more health problems (number of diagnoses> = 6) (54.2% vs 47.4%), but their utilization were actually lower, suggesting a combination of higher disease burden and lower healthcare use. These findings suggest that disparity may be masked if the uneven distribution of health risk factors of patients in rural and urban clinics is not considered. Even with a higher proportion of primary care clinics in rural areas in our study, patients in primary care clinics in rural areas had lower utilization than patients in primary care clinics in urban areas after adjusting for other factors. In specialty clinics, we observed a similar pattern of lower utilization among patients in rural clinics. This supports that a thorough evaluation of healthcare utilization is complex and requires consideration of multiple factors [34]. The phenomenon of lower healthcare utilization in rural areas could reflect geographical [24] or sociocultural barriers to accessing care in rural areas, issues with insurance coverage, time constraints, a relative lack of provider availability, or a lack of transportation [35]. With fewer healthcare encounters, rural patients may have less access to health information that could curtail health behaviors such as smoking, drinking, and lack of exercise, which are seen at higher incidences in rural areas [34]. There is also the possibility that rural clinics may operate more efficiently than urban clinics, meaning less visits noted, but similar health outcomes achieved. However, our findings show there is significant disparity in health status in patients in rural clinics when examining the association of patient characteristics visiting primary care clinics. In addition to the possible contributors to lower rural healthcare utilization discussed above, there could be differences in rural and urban clinic scheduling practices given lower provider availability in rural areas that contribute to less frequent encounters for patients in rural clinics.

Our findings argue for the critical importance to address barriers to healthcare with innovative sustainable solutions to limit rural disparity. One potential solution is to leverage the use of advancements in technology [36, 37] such as, telehealth and telecommunication [38, 39], health information technology, health management, decision support tools, patient education using outreach [40–42], and point of care models [5, 43].

To our knowledge, our study is unique in quantifying the healthcare utilization patterns of a diverse general adult patient population over a substantial study period using direct electronic medical record information. Previous data on factors associated with healthcare utilization have largely been driven by general population surveys, provider-based surveys, or Medicare claims information. Our data suggests that examining healthcare system level data yields similar conclusions for patients who access care. Even among patients with access to the same healthcare system, rural vs. urban clinic patients have worse health status and lower utilization, suggesting multiple factors (e.g., transportation) other than the general access to local healthcare may contribute to the overall health disparity.

Despite its novelty, there are limitations to this study. First, due to the restrictions on what factors are consistently reported or easily accessible in electronic health records, our analysis did not examine additional important factors such as insurance status, socioeconomic status, access to transportation, or family structure that could reasonably be associated with healthcare utilization. These results therefore need to be considered with caution. We suggest caution in interpreting these results that this study of patients engaged in a large healthcare system may suffer from an inherent selection bias that most patients accessing healthcare are likely to have insurance support. The impact of insurance on health status and utilization needs to be further studied. Second, using the number of diagnoses as a proxy for health status may not fully account for the severity of the individual diagnoses, which has important implications when determining one’s health status. Some of the quartiles also have a wide range of number of diagnoses, which might suggest a wide range of health statuses within a quartile. In secondary analysis, we reached similar results using either the number of diagnoses or Elixhauser comorbidity score as a proxy for patient overall health [29–31]. Third, this study categorized patients based on their clinic locations rather than personal addresses. To reduce the potential crossover (e.g, rural residing patients visiting an urban clinic), we evaluated patients who exclusively visit rural or urban clinics and excluded patients who visited both rural and urban clinics (only ~1% of the sample, suggesting that clinics largely served either a majority of patients living in rural areas or a majority of patients living in urban areas). The low crossover rate also reduces the concern of skewing results via the exclusion of rural patients needing specialty care requiring visits to urban clinics, which might reflect increased health needs and entail increased healthcare encounters among such patients. Lastly, although this study examines healthcare utilization patterns across large adult patient populations, we acknowledge these results may be limited in generalizability to other systems outside the greater St. Louis, southern Illinois, and mid-Missouri regions. However, given the sociodemographic, behavioral, and geographic diversity of the sample included in our study, we feel our findings can be informative on patterns of healthcare utilization to a wide range of healthcare systems.

In summary, our study adds to existing healthcare utilization research by quantifying the extent to which patients in rural clinics utilize healthcare differently than patients in urban clinics in the context of their predisposing characteristics, enabling factors, and health needs within a large healthcare system. In our study, patients in rural clinics had worse health status and lower healthcare utilization than patients in urban clinics, even within the same large healthcare system. Understanding the root causes of rural disparity is an important topic requiring further qualitative and quantitative research. The current work has the potential to guide future research on the association of rural healthcare system utilization with rural healthcare outcomes disparities and ways to address barriers to healthcare access once patients enter the healthcare system.

## Conclusions

Our research examining a large population of adults within a diverse healthcare system demonstrates lower outpatient healthcare utilization amongst rural populations despite worse health status amongst this group. This conclusion supports findings from previous healthcare utilization data which has largely been driven by general population surveys or Medicare claims information which might have a bias for healthier and older populations, respectively. Further studies exploring how unique healthcare utilization patterns among rural groups are related to health outcomes in other large scale healthcare systems can help generalize findings and identify possible solutions to eliminating rural healthcare disparities for patients who already access healthcare.

## Supporting information

S1 Table Distributions of clinic specialties differ in rural vs. urban clinics. (DOCX)

S2 Table Sample characteristics in rural vs. urban clinics. (DOCX)

S3 Table Healthcare utilization^a^ across patient characteristics including health professional shortage. (DOCX)

S4 Table Healthcare utilization^a^ across patient characteristics including Elixhauser. (DOCX)

S5 Table Association of patients characteristics and primary care utilization: Multivariate analyses^a,b^. (DOCX)

S6 Table Association of patients characteristics and specialty care utilization: Multivariate analyses ^a,b^. (DOCX)
