# Supplementary material for: Quantifying rural disparity in healthcare utilization in the United States: Analysis of a large midwestern healthcare system
Source: PLoS One. 2022 Feb 10;17(2):e0263718. doi: 10.1371/journal.pone.0263718 (PMC8830640; doi:10.1371/journal.pone.0263718)
Supplement: S1 Table — (DOCX) [file pone.0263718.s001.docx]

**S1 Table: Distributions of Clinic Specialties Differ in Rural vs. Urban Clinics**

| Clinics | *Overall* | | *Rural* | | *Urban* | |  |  |  |
| --- | --- | --- | --- | --- | --- | --- | --- | --- | --- |
|  | *n of clinics* | *%* | *n of clinics* | *(%)* | *n of clinics* | *(%)* | *ꭓ^2^* | *df* | *p** |
| **Primary Care Clinics** | | | | | | | | | |
| Family Medicine | 42 | 5.48 | 17 | 23.29 | 25 | 3.61 | 79.3 | 17 | <0.0001 |
| Pediatrics | 134 | 17.49 | 6 | 8.22 | 128 | 18.47 |  |  |  |
| Internal Medicine | 179 | 23.37 | 29 | 39.73 | 150 | 21.65 |  |  |  |
| **Specialty Clinics** | | | | | | |  |  |  |
| Ancillary Services | 44 | 5.74 | 6 | 8.22 | 38 | 5.48 |  |  |  |
| Dermatology | 11 | 1.44 | 0 | 0 | 11 | 1.59 |  |  |  |
| Neurological Surgery | 6 | 0.78 | 1 | 1.37 | 5 | 0.72 |  |  |  |
| Neurology | 40 | 5.22 | 1 | 1.37 | 39 | 5.63 |  |  |  |
| Obstetrics & Gynecology | 56 | 7.31 | 4 | 5.48 | 52 | 7.5 |  |  |  |
| Ophthalmology | 23 | 3 | 0 | 0 | 23 | 3.32 |  |  |  |
| Orthopedics & Sports Medicine | 66 | 8.62 | 1 | 1.37 | 65 | 9.38 |  |  |  |
| Otolaryngology | 21 | 2.74 | 2 | 2.74 | 19 | 2.74 |  |  |  |
| Pain Management | 8 | 1.04 | 1 | 1.37 | 7 | 1.01 |  |  |  |
| Physical Medicine & Rehabilitation | 12 | 1.57 | 0 | 0 | 12 | 1.73 |  |  |  |
| Psychiatry | 21 | 2.74 | 0 | 0 | 21 | 3.03 |  |  |  |
| Radiation Oncology | 8 | 1.04 | 0 | 0 | 8 | 1.15 |  |  |  |
| Radiology | 9 | 1.17 | 0 | 0 | 9 | 1.3 |  |  |  |
| Surgery | 78 | 10.18 | 5 | 6.85 | 73 | 10.53 |  |  |  |
| Urological Surgery | 8 | 1.04 | 0 | 0 | 8 | 1.15 |  |  |  |
| Total | 766 |  | 73 |  | 693 |  |  |  |  |
