# Supplementary material for: Quantifying rural disparity in healthcare utilization in the United States: Analysis of a large midwestern healthcare system
Source: PLoS One. 2022 Feb 10;17(2):e0263718. doi: 10.1371/journal.pone.0263718 (PMC8830640; doi:10.1371/journal.pone.0263718)
Supplement: S2 Table — (DOCX) [file pone.0263718.s002.docx]

**S2 Table: Sample Characteristics in Rural vs. Urban Clinics**

|  | Rural | Urban |  | | |  |
| --- | --- | --- | --- | --- | --- | --- |
|  | *n (%)* | *n (%)* |  |  |  |  |
|  | | | *ꭓ^2^* | *df* | **p* |  |
| Total | 50,250 (10.6%) | 424,424 (89.4%) |  |  |  |  |
| Age | | | | | |  |
| 18-49 | 19,322 (38.5%) | 152,244 (35.9%) | 306 | 3 | **<0.0001** |  |
| 50-59 | 8,948 (17.8%) | 82,131 (19.4%) |  |  |  |  |
| 60-69 | 10,064 (20.0%) | 95,558 (22.5%) |  |  |  |  |
| ≥70 | 11,916 (23.7%) | 94,491 (22.3%) |  |  |  |  |
| Gender^b^ | | | | | |  |
| Male | 20,432 (40.7%) | 171,765 (40.5%) | 0.551 | 1 | 0.4581 |  |
| Female | 29,817 (59.3%) | 252,466 (59.5%) |  |  |  |  |
| Race^c^ | | | | | |  |
| White | 47,115 (94.4%) | 324,093 (78.5%) | 7608 | 2 | **<0.0001** |  |
| Black | 1,384 (2.8%) | 71,191 (17.2%) |  |  |  |  |
| Other | 1,394 (2.8%) | 17,879 (4.3%) |  |  |  |  |
| Ethnicity^d^ | | | | | |  |
| Hispanic | 442 (0.9%) | 6,051 (1.5%) | 100.353 | 1 | **<0.0001** |  |
| Non-Hispanic | 46,992 (99.1%) | 393,680 (98.5%) |  |  |  |  |
| Smoking Status^e,f^ | | | | | |  |
| Non-smoker | 37,469 (79.3%) | 323,192 (86.1%) | 1504 | 1 | **<0.0001** |  |
| Smoker | 9,751 (20.7%) | 52,369 (13.9%) |  |  |  |  |
| Health Status | | | | | |  |
| Q1(≤2 diagnoses) | 13,610 (27.1%) | 130,092 (30.7%) | 935.1 | 3 | **<0.0001** |  |
| Q2 (3-5 diagnoses) | 9,439 (18.8%) | 93,356 (22.0%) |  |  |  |  |
| Q3(6-8 diagnoses) | 15,869 (31.6%) | 111,040 (26.2%) |  |  |  |  |
| Q4 (≥9 diagnoses) | 11,332 (22.6%) | 89,936 (21.2%) |  |  |  |  |
| ^a^ The sample was from Washington University School of Medicine/Barnes-Jewish Healthcare System of outpatients, serving the greater St. Louis, southern Illinois, and mid-Missouri regions from June 2018- March 2019. | | | | | | |
| ^b^ Documented gender was missing for 194 patients. | | | | | | |
| ^c^ Documented race was missing for 11,618 patients. | | | | | | |
| ^d^ Documented ethnicity was missing for 27,509 patients. | | | | | | |
| ^e^ Smoking status was missing for a total of 51,893 patients. | | | | | | |
| ^f^ These patients are unique and exclusively visited urban or rural clinics. | | | | | | |
| ^g^ Health Status is defined as the number of ICD 10 diagnosis codes by quartile. | | | | | | |
