# Supplementary material for: Quantifying rural disparity in healthcare utilization in the United States: Analysis of a large midwestern healthcare system
Source: PLoS One. 2022 Feb 10;17(2):e0263718. doi: 10.1371/journal.pone.0263718 (PMC8830640; doi:10.1371/journal.pone.0263718)
Supplement: S3 Table — (DOCX) [file pone.0263718.s003.docx]

**S3 Table: Healthcare Utilization^a^ Across Patient Characteristics including Health Professional Shortage**

|  | *RR*^b^ | *95% CI* | | *p** | |
| --- | --- | --- | --- | --- | --- |
| Age |  | | | | |
| 18-49 | Reference | | | | |
| 50-59 | 0.97 | 1.00, 1.02 | | **<0.0001** | |
| 60-69 | 0.99 | 1.04, 1.06 | |  |  |
| >=70 | 1.02 | 1.09, 1.10 | |  |  |
| Gender |  | | | | |
| Male | Reference | | | | |
| Female | 0.99 | 0.98, 1.00 | | 0.31 | |
| Race |  |  | |  | |
| White | Reference | | | | |
| Black | 1.00 | 0.97, 0.98 | | 0.19 | |
| Other | 0.98 | 0.92, 0.94 | |  |  |
| Ethnicity |  | | | | |
| Non-hispanic | Reference | | | | |
| Hispanic | 1.01 | 1.01, 1.06 | | 0.18 | |
| Smoking Status^c^ |  |  | |  | |
| Non-smoker | Reference | | | | |
| Smoker | 1.00 | 1.02, 1.03 | | 0.65 | |
| Health status^d^ |  |  |  |  |  |
| Q1​ (0-2) | Reference | | | | |
| Q2​ (3-5) | 1.69 | 1.46, 1.48 | | **<0.0001** | |
| Q3​ (6-8) | 2.42 | 1.98, 2.01 | |  |  |
| Q4​ (9-69) | 4.59 | 4.07, 4.13 | |  |  |
| Health professional shortage index (Clinician rate)^e^ |  |  |  |  |  |
| Low (<=600) | 0.94 | 0.89, 1.00 | | 0.11 | |
| Medium (601-900) | 0.99 | 0.95, 1.04 | |  |  |
| High (>900) | Reference | | | | |
| Location^f^ |  |  |  | | |
| Urban | Reference | | | | |
| Rural | 0.62 | 0.75, 0.76 | | **<0.0001** | |

^a^Healthcare Utilization is defined as number of visits to any outpatient clinics in 766 clinics serving the greater St. Louis, southern Illinois, and mid-Missouri regions from June 2018- March 2019.

^b^RR: Relative risk.

^c^Individuals were classified as smokers in this study if they were ever documented as a smoker in a clinic encounter recorded within the data timeframe. This identity was self-reported at the time of clinic encounter.

^d^Health Status is defined as the number of ICD 10 diagnosis codes by quartile.

^e^Clinician rate used in this application is calculated as clinician count divided by the respective population. Clinician rate is categorized into 3 levels:<=600, 601-900, and >900. The result then is multiplied by 100,000."

^f^These patients are unique and exclusively visited urban or rural clinics.

*Boldface indicates statistical significance (p<0.001).
