# Supplementary material for: Quantifying rural disparity in healthcare utilization in the United States: Analysis of a large midwestern healthcare system
Source: PLoS One. 2022 Feb 10;17(2):e0263718. doi: 10.1371/journal.pone.0263718 (PMC8830640; doi:10.1371/journal.pone.0263718)
Supplement: S4 Table — (DOCX) [file pone.0263718.s004.docx]

**S4 Table: Healthcare Utilization^a^ Across Patient Characteristics including Elixhauser**

|  | *RR*^b^ | *95% CI* | | *p** | |
| --- | --- | --- | --- | --- | --- |
| Age |  | | | | |
| 18-49 | Reference | | | | |
| 50-59 | 1.02 | 1.01, 1.04 | | **<0.0001** | |
| 60-69 | 1.08 | 1.07, 1.10 | |  |  |
| >=70 | 1.13 | 1.10, 1.15 | |  |  |
| Gender |  | | | | |
| Male | Reference | | | | |
| Female | 1.04 | 1.03, 1.05 | | **<0.0001** | |
| Race |  |  | |  | |
| White | Reference | | | | |
| Black | 0.99 | 0.97, 1.00 | | 0.0018 | |
| Other | 0.96 | 0.94, 0.98 | |  |  |
| Ethnicity |  | | | | |
| Non-hispanic | Reference | | | | |
| Hispanic | 1.02 | 0.98, 1.05 | | 0.33 | |
| Smoking Status^c^ |  |  | |  | |
| Non-smoker | Reference | | | | |
| Smoker | 1.03 | 1.02, 1.04 | | **0.0001** | |
| Health status (Elixhauser Index)^d^ |  |  |  |  |  |
| 0 | Reference | | | | |
| 1 | 1.46 | 1.40, 1.51 | | **<0.0001** | |
| 2+ | 2.26 | 2.15, 2.38 | |  |  |
| Location^e^ |  |  |  | | |
| Urban | Reference | | | | |
| Rural | 0.54 | 0.44, 0.66 | | **<0.0001** | |

^a^Healthcare Utilization is defined as number of visits to any outpatient clinics in 766 clinics serving the greater St. Louis, southern Illinois, and mid-Missouri regions from June 2018- March 2019.

^b^RR: Relative risk.

^c^Individuals were classified as smokers in this study if they were ever documented as a smoker in a clinic encounter recorded within the data timeframe. This identity was self-reported at the time of clinic encounter.

^d^Health Status is defined by the Elixhauser Index with 3 levels: 0, 1, and 2+.

^e^These patients are unique and exclusively visited urban or rural clinics.

*Boldface indicates statistical significance (p<0.001).
