# Supplementary material for: Quantifying rural disparity in healthcare utilization in the United States: Analysis of a large midwestern healthcare system
Source: PLoS One. 2022 Feb 10;17(2):e0263718. doi: 10.1371/journal.pone.0263718 (PMC8830640; doi:10.1371/journal.pone.0263718)
Supplement: S5 Table — (DOCX) [file pone.0263718.s005.docx]

**S5 Table: Association of Patients Characteristics and Primary Care Utilization: Multivariate Analyses**^a,b^

|  | *RR*^c^ | *95% CI* | | *p** |
| --- | --- | --- | --- | --- |
| Age |  | | | |
| 18-49 | Reference | | | |
| 50-59 | 0.97 | 0.96, 0.99 | | **0.0002** |
| 60-69 | 1.02 | 1.00, 1.03 | |  |
| ≥70 | 1.10 | 1.07, 1.12 | |  |
| Gender |  | | | |
| Male | Reference | | | |
| Female | 1.02 | 1.01, 1.03 | | **0.0007** |
| Race |  |  | |  |
| White | Reference | | | |
| Black | 0.99 | 0.98, 1.00 | | 0.0027 |
| Other | 0.94 | 0.91, 0.97 | |  |
| Ethnicity |  |  |  |  |
| Non-Hispanic | Reference |  | |  |
| Hispanic | 1.01 | 0.98, 1.04 | | 0.45 |
| Smoking Status^d^ |  |  | |  |
| Non-smoker | Reference | | | |
| Smoker | 0.99 | 0.98, 1.01 | | 0.51 |
| Health Status^e^ |  |  |  |  |
| Q1(≤2 diagnoses) | Reference |  |  | **<0.0001** |
| Q2 (3-5 diagnoses) | 1.58 | 1.45, 1.73 | |  |
| Q3 (6-8 diagnoses) | 2.18 | 1.99, 2.39 | |  |
| Q4 (≥9 diagnoses) | 5.01 | 4.57, 5.50 | |  |
| Location^f^ |  |  |  | |
| Urban | Reference | | | |
| Rural | 0.66 | 0.58, 0.74 | | **0.0003** |
| ^a^ Family Medicine, Internal Medicine and Pediatrics clinics were defined as primary care clinics. All other clinics were defined as Specialty clinics. | | | | |
| ^b^ Healthcare Utilization is defined as number of visits to any outpatient clinics in 766 clinics serving the greater St. Louis, southern Illinois, and mid-Missouri regions from June 2018- March 2019. | | | | |
| ^c^ RR: Relative risk. | | | | |
| ^d^ Individuals were classified as smokers in this study if they were ever documented as a smoker in a clinic encounter recorded within the data timeframe. This identity was self-reported at the time of clinic encounter. | | | | |
| ^e^ Health Status is defined as the number of ICD 10 diagnosis codes by quartile. | | | | |
| ^f^ These patients are unique and exclusively visited urban or rural clinics. | | | | |
| *Boldface indicates statistical significance (p<0.001). | | | | |
